# Promoting Articular Cartilage Regeneration through Microenvironmental Regulation

**DOI:** 10.1155/2024/4751168

**Published:** 2024-07-26

**Authors:** Kai Liu, Bingjun Zhang, Xiaoling Zhang

**Affiliations:** ^1^ Department of Orthopedic Surgery Xin Hua Hospital Affiliated to Shanghai Jiao Tong University School of Medicine, Shanghai 200092, China; ^2^ Collaborative Innovation Centre of Regenerative Medicine and Medical BioResource Development and Application Co-constructed by the Province and Ministry Guangxi Medical University, Nanning, Guangxi 530021, China

## Abstract

In recent years, as the aging population continues to grow, osteoarthritis (OA) has emerged as a leading cause of disability, with its incidence rising annually. Current treatments of OA include exercise and medications in the early stages and total joint replacement in the late stages. These approaches only relieve pain and reduce inflammation; however, they have significant side effects and high costs. Therefore, there is an urgent need to identify effective treatment methods that can delay the pathological progression of this condition. The changes in the articular cartilage microenvironment, which are complex and diverse, can aggravate the pathological progression into a vicious cycle, inhibiting the repair and regeneration of articular cartilage. Understanding these intricate changes in the microenvironment is crucial for devising effective treatment modalities. By searching relevant research articles and clinical trials in PubMed according to the keywords of articular cartilage, microenvironment, OA, mechanical force, hypoxia, cytokine, and cell senescence. This study first summarizes the factors affecting articular cartilage regeneration, then proposes corresponding treatment strategies, and finally points out the future research direction. We find that regulating the opening of mechanosensitive ion channels, regulating the expression of HIF-1, delivering growth factors, and clearing senescent cells can promote the formation of articular cartilage regeneration microenvironment. This study provides a new idea for the treatment of OA in the future, which can promote the regeneration of articular cartilage through the regulation of the microenvironment so as to achieve the purpose of treating OA.

## 1. Introduction

Articular cartilage is a physiologically non-self-renewing avascular tissue. It comprises a single cell type, chondrocytes, which are responsible for the synthesize and secretion of matrix and fibers [1]. Enclosed within the cartilaginous stroma, chondrocytes reside in a small cavity called the cartilaginous lacuna. Osteoarthritis (OA) is a multifactorial condition that can be attributed to various factors, including age, heredity, obesity, and trauma.

Injury to the articular cartilage can lead to excessive osteoclast absorption and abnormal remodeling of the subchondral bone [2]. Consequently, articular cartilage lesions result in subchondral bone thickening, osteophyte formation, synovial inflammation, ligament degeneration, and capsule hypertrophy [3]. These changes represent the typical pathological features observed in OA.

OA is a prevalent degenerative disease and a significant cause of disability worldwide, affecting more than 303 million individuals [4]. As the global population ages, the incidence of OA is on the rise. Current treatment methods involve exercise and drug therapy in the early stages, progressing to joint replacement in the advanced stages. However, these treatments address pain and inflammation without promoting tissue repair and regeneration. Additionally, the effectiveness of drug therapy is limited due to poor drug penetration caused by the lack of blood vessels in joints [5]. The drug is quickly discharged from the joint cavity through synovial capillaries and lymphatic drainage, and the stay time in the joint is as short as 1–4 hr. What little drug remains within the joint is excluded from the chondrocytes by the spatially dense cartilage extracellular matrix (ECM) [6]. Given the inadequacy of current treatment for most patients, there is an urgent need for more effective solutions. Most scientists focus on cartilage regeneration, aiming to develop new tissue with the same physiological characteristics as natural cartilage. The ultimate goal is to replace the diseased cartilage tissue with regenerated tissue, offering a promising avenue for future OA treatment. The most crucial aspect of cartilage regeneration is the application of stem cells because of their feasibility of cell expansion and differentiation *in vitro*. Mesenchymal stem cells (MSCs) hold significant promise in the cellular treatment of cartilage defects owing to their capacity for chondrogenesis [7]. Exosomes secreted by stem cells play immunomodulatory and anti-inflammatory roles in the inflammatory microenvironment [8], mainly through the polarization of M2 macrophages, thereby reducing inflammation [9].

The advancement of tissue engineering offers a novel approach to promoting articular cartilage regeneration, with biomaterials playing a pivotal role in this progress. Among these biomaterials, hydrogels have shown significant advantages in studying cartilage regeneration and its pathological mechanisms. DNA hydrogels have demonstrated efficacy in drug delivery applications with their favorable histocompatibility, degradability, design, and regulation [10]. Nanoparticles (NPs) have been widely used in the field of biomaterials due to their expedited synthesis duration and low cost [11]. Those synthesized by thermophilic bacteria are considered environmentally friendly and hold promise as alternatives to prevailing chemical and physical synthesis methods [12]. Recent studies highlight the extensive use of nanofiber scaffolds in articular cartilage repairs. These scaffolds can stimulate the regeneration of new tissues and ECM components and exhibit good histocompatibility [13]. Silk fibroin (SF) is a polymer material used in cartilage repair treatment due to its commendable mechanical properties and degradability [14].

Numerous studies have substantiated using biomaterials to treat OA and promote cartilage regeneration. However, articular cartilage regeneration is affected by many factors, the most important of which is the articular cartilage microenvironment. The inflammatory environment caused by cartilage injury is a pivotal factor leading to the death and hypertrophy of chondrocytes, ECM destruction, and ectopic bone formation. However, there exists a gap in the literature regarding which factors in the articular cartilage microenvironment accelerate the occurrence and development of OA. Furthermore, the therapeutic strategies targeting the articular cartilage microenvironment remain unexplored. Cellular senescence, characterized by the expression of a cellular senescence phenotype and the secretion of pro-inflammatory factors into the microenvironment, inhibits cartilage regeneration [15]. Conversely, optimal stimulation of the cartilage microenvironment promotes cartilage regeneration through mechanical force, partial pressure of oxygen, and cytokines.

## 2. Classification of Articular Cartilage

At birth, the articular cartilage is a dense, highly cellular tissue. While the chondrocytes are isotropic, the distribution of ECM is notably poor. With the maturation and formation of articular cartilage before early adulthood, chondrocytes in different regions receive varying degrees of mechanical, electrical, and physicochemical signals from the articular cartilage. This results in an increase in cell size, ECM accumulation, and compositional changes, particularly alterations in collagen composition, ultimately leading to the development of zonal anisotropic tissue and the formation of three distinct types of articular cartilage [16].

The three types of articular cartilage are hyaline, fibrotic, and elastic. Hyaline cartilage is the most common type found at the junction of the human rib and sternum and on the joint surface of the trachea and synovial joints, such as the elbow and knee joints [17]. Fibrocartilage is mainly found in structures like intervertebral discs, meniscus, bone-tendon, and ligament-tendon interfaces. It contains a high density of type I collagen, providing high tensile strength [18]. Elastic cartilage is distributed in the auricle and epiglottis. Its structural feature is characterized by many intertwined elastic fibers in the matrix, with denser fibers observed in the middle of the cartilage [19].

## 3. Structure and Physiology of Articular Cartilage in Normal and Osteoarthritic Joints

**Figure 1:**
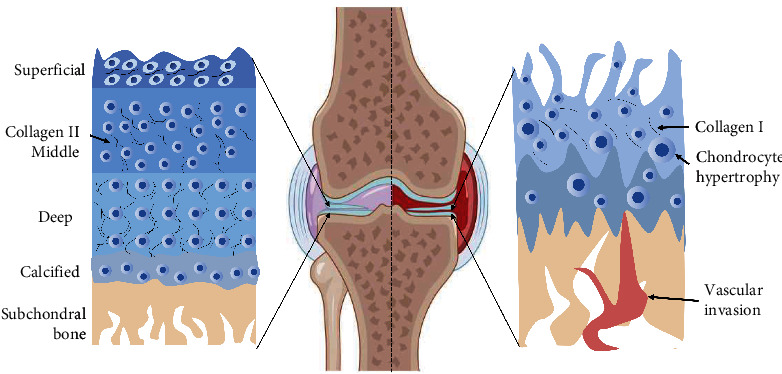
Microstructure and pathological changes of normal and damaged cartilage. Articular cartilage is divided into surface layer, middle layer, deep layer, calcified layer, and subchondral bone [20]. Normal cartilage is rich in type II collagen and ECM. When cartilage is damaged, it is usually repaired into fibrocartilage, which is rich in type I collagen. The internal manifestations of the damaged cartilage are chondrocyte hypertrophy and vascular invasion.

Articular cartilage is well-recognized for its stratification into distinct layers: the surface layer, middle layer, deep layer, calcified layer, and subchondral bone [20] (Figure 1). Its primary function is to minimize the stress generated during joint loading, enabling normal joints to withstand significant forces during movement. Literature characterizes articular cartilage as a composite solid organic matrix saturated with water and mobile ions [21]. The critical components of this matrix are collagen molecules and negatively charged proteoglycans. Collagen can aggregate into fibers of different sizes that vary with the depth of the cartilage layer [22, 23]. Proteoglycans within articular cartilage are biomolecules consisting of a protein core attached to numerous glycosaminoglycan side chains with a negatively charged moiety [24, 25]. The collagen fibers in the most superficial areas of the cartilage layer are densely arranged and parallel to the joint surface. This collagen, characterized by low proteoglycan content and low permeability, and is able to act as a resistance to the high resistance to fluid movement when the cartilage is compressed [26]. In the middle layer, collagen fibers are larger and randomly arranged, with an increased proteoglycan composition resulting in high pressure and water content [27]. In the deepest layer, the proteoglycan content is decreased, and the collagen fibers are larger, forming bundles perpendicular to the calcification/bone interface [28]. Zhang et al. [29] provided detailed insights into changes in gene transcription in chondrocyte subsets during the pathology of OA. Through single-cell RNA sequencing, they identified a cluster with high expression of *sox9*, *acan*, *comp*, and *fn* in the chondrocyte population, which they subdivided into three clusters (chondrocytes 1 (C1), C2, and C3). They reviewed the literature and found that *mmp2*, *col14a1*, and *col22a1* were mainly expressed in the superficial region and were highly enriched in the C1 or C2 clusters [30, 31, 32]. *Cytl1* was highly enriched in the C3 cluster and expressed only in the intermediate region. Therefore, they inferred that clusters C1 and C2 represented superficial chondrocytes, while cluster C3 represented chondrocytes in the intermediate zone [29].

Changes in the cartilage in OA are mainly related to the imbalance in tissue remodeling caused by the behavior of chondrocytes [33]. Articular hyaline cartilage, a viscoelastic nonvascular connective tissue, possesses a smooth surface, antifriction, and load-bearing properties [34]. It is a structured network of dense ECM, housing highly differentiated chondrocytes with low metabolic activity, enabling survival under hypoxic conditions. The ECM primarily comprises water, collagen (mainly type II collagen fibers), proteoglycans (mainly collagen polysaccharides), and other noncollagen proteins (connexin, fibronectin, and cartilage oligomeric matrix proteins) [35].

In OA, the joint surface typically experiences swellings, and as fibrillation progresses, an intact layer of erosion exposes the subchondral bone [36]. Chondrocytes contribute to ECM damage by producing ECM-degrading enzymes, such as matrix metalloproteinases (MMPs). These enzymes regulate the expression of several cytokines, chemokines, inflammatory mediators, and matrix-degrading enzymes [37]. A key characteristic of OA is increased chondrocyte proliferation and the production of inflammatory mediators such as interleukin 1*β* (IL-1*β*) [38], interleukin 6 (IL-6) [39], tumor necrosis factor-*α* (TNF-*α*) [40], reactive oxygen species (ROS), and nitric oxide (NO) [41]. These factors accelerate chondrocyte apoptosis and the senescence of intra-articular cells. Senescent cells secrete elevated levels of bioactive molecules such as chemokines, cytokines, proteases, and growth factors [42], which can induce inflammation in the periarticular cartilage, further aggravating articular cartilage injury and chondrocyte apoptosis.

## 4. Microenvironmental Factors Influencing Cartilage Regeneration and Treatment Strategies for OA

Chondrocyte metabolism and cartilage homeostasis largely depend on regulating nutrients, oxygen, and other factors within the subchondral bone and synovial fluid matrix. Several approaches in the treatment of OA have been found to generate cartilage with properties similar to natural cartilage and capable of performing unique functions. However, these efforts yielded unsatisfactory results due to the avascular nature of cartilage, which hampers cell penetration and thus limits the repair process [43].

**Figure 2:**
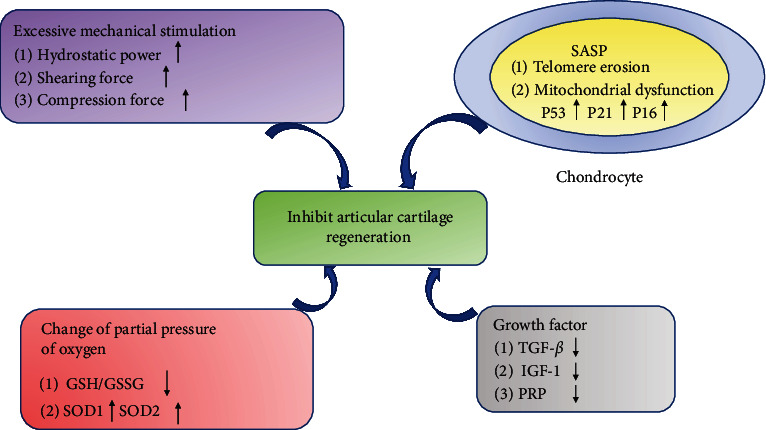
Microenvironmental factors affecting cartilage regeneration. Articular cartilage regeneration is affected by many factors. Excessive mechanical stress stimulation, such as hydrostatic force, shear force, and compression force [50]. Some cytokines are produced by senescent cells, such as IL-6, IL-17, and TNF-*α* [51]. The change of oxygen partial pressure resulted in the decrease of glutathione (GSH)/oxidized glutathione (GSSG) ratio and the increase of superoxide dismutase 1 (SOD1) and superoxide dismutase 2 (SOD2) protein expression [52]. The decrease of growth factors such as transforming growth factor-*β* (TGF-*β*), insulin-like growth factor-1 (IGF-1), and platelet-rich plasma (PRP) [53]. All these factors inhibit the regeneration of cartilage.

The interactions within the microenvironment are complex and diverse [44], inducing a series of physiological responses through various signaling pathways and the release of cytokines. Consequently, microenvironment regulation has emerged as a critical factor influencing articular cartilage regeneration. This section discusses the known factors regulating cartilage homeostasis and function within the chondrocyte microenvironment, mainly focusing on mechanical stimulation [45], oxygen partial pressure [46], cytokines [47], alterations in immune cells [48], and cell senescence [49] following cartilage damage (Figure 2). Additionally, we outline strategies for treating OA based on regulating the articular cartilage regeneration microenvironment.

### 4.1. Mechanical Stimulation and Mechanosensitive Ion Channel

Moderate mechanical stress induces cartilage formation during fetal development and maintains the dynamic balance of adult cartilage [54]. However, the significant impacts of falls, sports injuries, and traffic accidents can cause cartilage damage. Research indicates that impact force can permanently change the mechanical properties of articular cartilage beyond a certain threshold and disrupt its structural integrity [55, 56]. In addition, repeated heavy loads have been shown to induce mitochondrial dysfunction in chondrocytes, reduce adenosine triphosphate levels, and aggravate proton leakage and ROS formation [57]. TGF-*β* signal transduction is recognized as a critical mediator of cartilage regulation in response to mechanical stress. A recent study demonstrated that TGF-*β* activity was concentrated in cartilage areas experiencing high mechanical stress, which could damage the metabolic activity of chondrocytes and destroy cartilage homeostasis [58]. Various pathways, including MAPK-ERK, Wnt, and Hedgehog signaling, also regulate the mechanical transduction of chondrocytes [54]. Different intensities of mechanical stress exert different effects on the microenvironment of articular cartilage, thereby playing various roles in articular cartilage regeneration.

Insights into mechanical biology offer new strategies for regenerative medicine. Mechanical stimulation can be introduced into the expansion culture of cell-based therapy to replicate the natural environment of articular cartilage. At the cellular level, biomechanics have been shown to effectively enhance the cartilage formation of MSCs, which may be attributed to the increase of cartilage formation signals mediated by TGF-*β* [59, 60]. Furthermore, low levels of shear stress can stimulate the migration of JNK and p38MAPK pathways mediated by human MSCs through the SDF-1/CXCR4 axis [61]. Additionally, mechanical loading enhances angiogenesis, which can be attributed to a cascade of fibroblast growth factor receptor and vascular endothelial growth factor receptor signals [62]. Cartilage is primarily controlled by mechanical forces such as hydrostatic, shear, and compressive forces. Dynamic tissue shear and periodic uniaxial stress have been shown to enhance the integrity of the cartilage matrix [50]. Evidence suggests that periodic hydrostatic pressure can induce the corresponding intrinsic immune response to Piezo1. Piezo1 is a mechanically gated ion channel of intrinsic immune cells that upregulates the expression of pro-inflammatory genes [63].

Mechanical factors play an essential role in the articular cartilage microenvironment and contribute to the occurrence and development of OA. Mechanical stimulation profoundly affects cartilage maintenance, degeneration, and regeneration through the mechanical transduction of chondrocytes. Therefore, understanding how mechanical conduction is transmitted in chondrocytes is essential for treating OA [64]. Chondrocytes are mechanically sensitive because they express many mechanically activated ion channels, including Piezo1, Piezo2, and transient receptor potential vanilloid 4 (TRPV4) [65, 66, 67], which represent potential therapeutic targets for promoting articular cartilage regeneration and treating OA.

**Figure 3:**
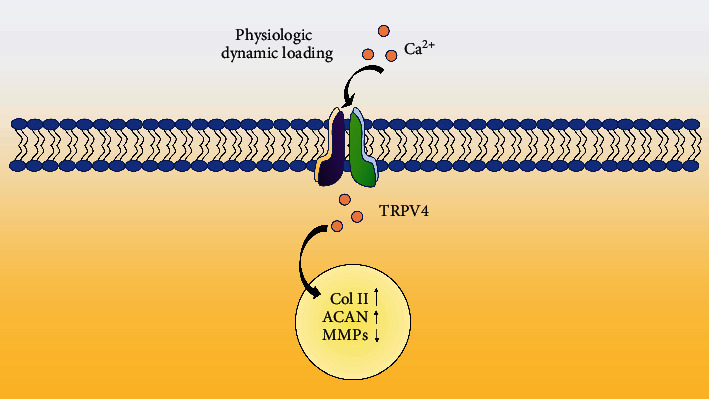
TRPV4-mediated mechanotransduction. Under physiological dynamic load, the TRPV4 ion channel is activated, Ca^2+^ influx promotes the expression of type II collagen (Col II) and aggrecan (ACAN) and reduces the expression of matrix metalloproteinases (MMPs) [64].

TRPV4 is a cationic channel that facilitates Ca^2+^ influx, vital for sensing mechanical stimulation and triggering chondrocyte anabolism. TRPV4 is sensitive to osmotic pressure, and its activation is caused by osmotic stress stimulation transmitted by mechanical loads (Figure 3). In a phase I clinical trial, the TRPV4 antagonist GSK2798745 was well tolerated in healthy volunteers without causing adverse reactions [68]. Additionally, studies implicated TRPV4 in the causation of various diseases, such as neuropathic pain, edema, gastrointestinal diseases, lung diseases [69], and OA [70]. Therefore, the research on TRPV4 has a broad biomedical application prospect. TRPV4 channels have been proven to affect chondrocyte differentiation under physiological loads, promoting intracellular Ca^2+^ influx and the expression of *sox9* and type II collagen [71]. TRPV4 is an essential factor leading to chondrocyte apoptosis in response to excessive mechanical stimulation (20% stretch) [72]. This is because TRPV4 activation is usually caused by changes in osmotic pressure, resulting in excessive physiological pressure within chondrocytes, ultimately causing chondrocyte death.

**Figure 4:**
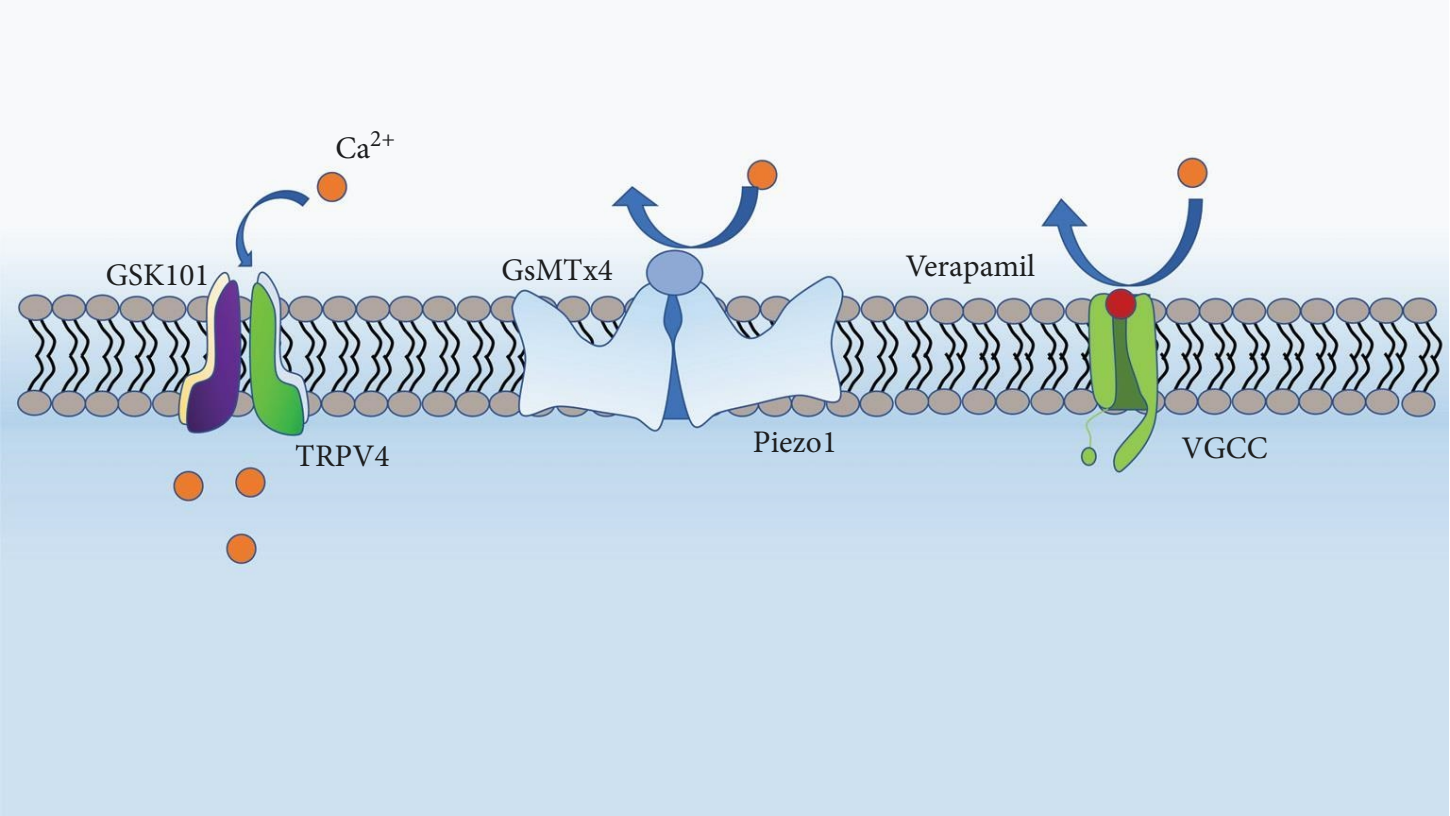
Therapeutic strategies for mechanical stimulation. GSK101 activates the TRPV4 channel, which in turn enhances the expression of type II collagen and aggrecan [74]. GsMTx4 inhibits the opening of Piezo1 channels, and Verapamil inhibits VGCC channels, which together regulate ca^2+^ homeostasis under abnormal load, thereby protecting chondrocytes [64].

The importance of TRPV4 in cartilage health has also been highlighted *in vivo* experiments. Compared to controls, TRPV4 knockout accelerated the development of aging-related OA [73]. Studies have shown that using a TRPV4 agonist (GSK101) increases type II collagen and glycosaminoglycan sulfate (GAG) in the cartilage when the physiological load is defined as a 10% strain [74]. In contrast, using a TRPV4 (GSK205) antagonist with mechanical load decreased type II collagen expression while increasing MMPs in the cartilage [75]. This suggests that TRPV4 activation may be a strategy to promote articular cartilage regeneration and effectively treat OA within the OA microenvironment (Figure 4).

Piezo1 is abundantly expressed in many tissues, including the lungs, colon, bladder, kidneys, and blood vessels [66]. Piezo1 channels are activated in response to external mechanical forces, activating intracellular signaling pathways that regulate cell volume or remodel host tissues. Mutations in *piezo1* and *piezo2* genes can lead to various human genetic diseases. A loss-of-function mutation in the *piezo1* gene can result in congenital lymphatic dysplasia. Similarly, a loss-of-function mutation in the *piezo2* gene can cause muscular dystrophy syndrome with joint curvature and scoliosis [76]. Piezo1 and Piezo2 also play important roles in bone development and bone homeostasis induced by mechanical stimulation [77]. Piezo2 channels are highly expressed in sensory-mechanical sensors in the sensory system and Merkel cells, which control limb movements and touch [78].

Treatment of chondrocytes with IL-1*α* can activate IL-1R and increase the expression of *piezo1* [79]. The enhancement of *piezo1* can increase the sensitivity of chondrocytes to mechanical stimulation and increase Ca^2+^ influx. IL-1*α*-treatment preferentially increases Piezo1 channels of primary articular chondrocytes but does not increase Piezo2 or TRPV4 channels. Atomic force microscopy-based assay data reveal the increased Ca^2+^ influx from cyclic physiologic loading in IL-1*α*-treated or Yoda1 (a Piezo1-specific agonist) chondrocytes compared to controls [64]. These data suggest that Piezo1 participates in the inflammatory response, disrupts Ca^2+^ homeostasis, and increases the mechano-sensitivity of chondrocytes to mechanical loads. Experiments have shown that knocking out *piezo2* in inflamed mice reduces their sensitivity and pain response to tactile stimuli, suggesting that *piezo2* plays an important role in pain perception during inflammation [80]. Although the functions of Piezo1 and Piezo2 have been outlined, there are currently no clinical trials on drugs targeting *Piezo1* and *Piezo2*. Hence, expediting drug research on *Piezo1* and *Piezo2* and their application holds significant promise.

**Figure 5:**
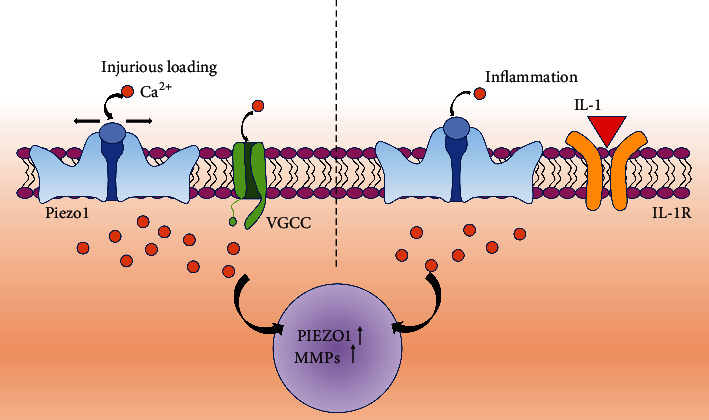
Piezo1-mediated mechanotransduction. Under the stimulation of excessive mechanical load and IL-1*α*, ca^2+^ enters the cells through the Piezo1 channel, increasing the expression of Piezo1 and MMPs and increasing the mechanical sensitivity of chondrocytes to mechanical loads [81].

Since Piezo1 and Piezo2 were identified as mechanically sensitive cation channels in 2010, researchers have explored the physiological responses induced by mechanical forces (Figure 5). Articular chondrocytes strongly express Piezo1 and Piezo2; both channels detect mechanical loads at the level of injury, particularly high strain. Knockout of *piezo1* and *piezo2* genes or treatment with piezo1 and piezo2 inhibitors GsMTx4 can reduce Ca^2+^ influx in chondrocytes [64]. Studies have shown that after IL-*1α* treatment of articular chondrocytes, Piezo1 channels are activated, while Piezo2 and TRPV4 channels are not, indicating that Piezo1 plays a role in inflammation. Additionally, Gao et al. [64] found that the activation of Piezo1 may lead to the activation of VGCC (inhibitors of L-type voltage-gated Ca^2+^ channels), which amplifies the perceptual signal of mechanical stimulation. Verapamil is a Ca^2+^ channel blocker that can reduce the changes in Ca^2+^ levels caused by injury. It has been proved that Verapamil could decrease the Wnt/*β*-catenin signal in patients with OA, thus downregulating the catabolism of ECM [64]. GsMTx4 inhibits the protective effect of Piezo1/2 on the cartilage, as confirmed in a cartilage explant injury model [82]. Transplanted cartilage pre-incubated with GsMTx4 significantly reduced the injury and death of chondrocytes after *in vivo* tissue injury.

Sun et al. [83] found that the G protein-coupled estrogen receptor inhibits the mechanical stimulation-mediated RhoA pathway and actin polymerization by promoting the expression and nuclear localization of YAP, thereby inhibiting the expression of *piezo1* and protecting cartilage. Lawrence et al. [84] found that endogenous peptides (Ucn1) could inhibit the ion channels of Piezo1, thereby delaying the progression of arthritis. These suggest that inhibition of mechanical transduction mediated by *Piezo1* and *Piezo2* is beneficial for cartilage anabolism, reduces cartilage catabolism, and promotes cartilage regeneration (Figure 4).

### 4.2. Change of Partial Pressure of Oxygen and Hypoxia Regulation

Oxygen tension in cartilage is greatly affected by many factors, including oxygen concentration in synovial fluid, cartilage thickness, cell density, and cell oxygen consumption rate, as well as oxygen supply to subchondral bone [85]. However, the oxygen tension of cartilage changes after injury. Chondrocytes that survive under deep and hypoxic pressure may encounter oxygen-rich synovial fluid because of cartilage injury. With the development of OA, the material transport of cartilage is accelerated by an increase of blood vessels [86], which leads to damaged cartilage under hyperoxia. Moderate partial pressure of oxygen is beneficial, but a partial pressure of oxygen that is either too low or too high is not conducive to the chondrogenesis of chondrocytes. Normoxic partial pressure promotes the dedifferentiation of chondrocytes to MSCs phenotype and impairs chondrogenesis [87]. TNF-*α* can increase the production of NO and PGE2 and the activity of MMPs at 5% oxygen partial pressure, indicating that 5% oxygen partial pressure is harmful to cartilage repair in the case of inflammation [88].

A change in oxygen partial pressure will also affect the energy metabolism and redox system of chondrocytes [89]. A partial pressure of oxygen that is too low significantly decreases cell viability [90], decreases the ratio of glutathione (GSH) to oxidized glutathione (GSSG), increases the protein expression of superoxide dismutase 1 (SOD1) and superoxide dismutase 2 (SOD2), increases glycosaminoglycan release, and changes the mitochondrial membrane potential and level of ROS [52].

It is known that when tissue oxygen concentration changes, it causes a series of intracellular responses, including regulation of angiogenesis, cell growth, metastasis, and other processes [91]. As such, controlling oxygen concentration may play a role in maintaining healthy articular cartilage. Articular cartilage is a kind of hypoxic tissue, and its oxygen partial pressure gradient decreases gradually from the surface to the deep layer of the cartilage. When articular cartilage is damaged, its oxygen partial pressure gradient will change. In OA, the oxygen consumption of synovium increases, but the ability to transport oxygen weakens, resulting in a lower partial pressure of oxygen than in healthy persons [92]. Some studies have suggested that hypoxia plays a role in the differentiation of MSCs into cartilage. The oxygen concentration of articular cartilage *in vivo* is between 1% and 4%. The results of *in vitro* simulation experiments show that this range of oxygen concentration is conducive to MSCs proliferation and cartilage formation and can inhibit cartilage hypertrophy [93].

HIF-1*α* is an amino acid polypeptide encoded by the HIF1A gene located on chromosome 14 that controls the oxygen balance in the cell [94, 95]. HIF-1*α* is ubiquitous in human and mammalian cells and is expressed under normoxia. However, the synthesized HIF-1*α* protein is quickly degraded by the intracellular oxygen-dependent ubiquitin protease degradation pathway, and HIF-1*α* can be stably expressed only under hypoxia. Under hypoxic conditions, HIF-1*α* accumulates in the cytoplasm because its hydroxylation is inhibited; it then phosphorylates and transfers to the nucleus, forming a dimer with HIF-*β* to bind to hypoxia response elements on hypoxia-sensitive genes, such as *vegf*, *sox9*, *col II*, etc. [96, 97]. The transcription of these target genes regulated by HIF-1*α* may play a protective role in articular cartilage damage [98]. Grimmer et al. [99] demonstrated that HIF-1*α* could be stably expressed under hypoxia and could enhance the accumulation of type II collagen in articular chondrocytes, but the effect was inhibited by the addition of an HIF-1*α* inhibitor. It has been reported that hypoxia could regulate the activity of HIF-1 at many levels, and it was considered to a key transcription factor for many cells and tissues to induce changes in related gene expression under hypoxia conditions. A recently published study demonstrated that HIF-1 can protect the metabolism of articular cartilage by inhibiting the signal of NF-*κ*B and the expression of MMP13 [100]. Therefore, regulating the expression level of the HIF-1 gene in the microenvironment of articular cartilage via oxygen partial pressure changes may function as a therapeutic strategy for articular cartilage repair and regeneration.

### 4.3. Growth Factors and Delivery

Cartilage formation is stimulated by cytokines and growth factors, such as TGF-*β* [101] and IGF-1 [53]. Maintaining these factors within normal ranges is crucial for ensuring cartilage growth and regeneration. Bone morphogenetic protein-2-mediated (BMP-2) differentiation of MSCs into chondrocytes has been proven to occur through the TGF-*β*3 pathway. BMP-6 has also been shown to promote chondrogenesis of bone marrow MSCs and stimulate matrix synthesis [102, 103]. Among natural growth factors, PRP has been extensively studied. In some studies, PRP was used as the primary treatment, such as in the study by Sánchez et al. [104], who treated severe knee OA by combining intra-articular injection with PRP intraosseous infiltration, reulting in improvement in joint function and pain relief according to knee injury and OA outcome scores (KOOS).

TGF-*β* is expressed at high levels in normal cartilage but is almost absent in OA cartilage [105]. Blocking TGF-*β* renders cartilage more susceptible to damage [106]. TGF-*β* is crucial for cartilage ECM synthesis. Thus, its absence leads to decreased ECM deposition and increased catabolic activity. TGF-*β* is an important factor in maintaining the balance of anabolism and catabolism in OA, suggesting its potential as a future treatment for OA. TGF-*β* deficiency can lead to embryonic death in mice, and survivors may experience severe inflammation [107, 108]. TGF-*β* deficient mice exhibit structural defects in bones, characterized by joint relaxation. In chondrocytes, interfering with TGF-*β* signal transduction will adversely affect osteocyte differentiation and aggravate the development of OA. Therefore, supplementing TGF-*β* can help maintain or repair cartilage. The injection of TGF-*β* into mice with arthritis has been shown to stimulate the synthesis of proteoglycans [109]. In an animal experimental study, rats with OA were treated with exercise, and it was found that TGF-*β* was upregulated, increased the number of chondrocytes, alleviated OA, and improved the quality of life of rats [110]. A study confirmed that injection of TGF-*β*1 expressing chondrocytes into the articular cavity of rabbits could significantly inhibit cartilage matrix degradation and improve tissue structure and collagen deposition, thus inhibiting articular cartilage degeneration and promoting its repair in OA [111]. Grimoud et al. [112] suggested that TGF-*β* gene transfer may provide a potential therapeutic strategy for the treatment of OA.

The articular cartilage lacks blood vessels, making nutrient supply challenging and self-repair difficult upon damage. Traditional treatment involves surgery, which comes with significant disadvantages. In normal articular cartilage, there is a delicate balance between anabolism and catabolism. Even with elevated catabolism, sufficient anabolism compensation can prevent the occurrence of OA. In OA, some factors break down cartilage and factors that help in synthesizing it, such as TGF-*β*, which synthesizes ECM [113]. Some studies have proved that the increase of anabolism of OA in the early stage was due to the upregulation of TGF-*β* expression [114]. TGF-*β* regulates cell proliferation, differentiation, apoptosis, and migration and can mediate the response of cells and tissues to injury [115]. Deficiency of TGF-*β* or abnormal signal transduction will lead to the phenotype of articular cartilage consistent with the pathological morphology of articular cartilage in OA. Reduced TGF-*β* receptor and TGF-*β* signal have been observed in aged OA mice. Inhibition of the expression of TGF-*β* in cartilage can aggravate the damage to articular cartilage [106]. TGF-*β* stimulates chondrocytes to produce more proteoglycans and type II collagen [109]. Advances in biomaterials, especially 3D printing technology, have sparked research into the delivery of anti-inflammatory cytokines to the articular cartilage microenvironment using biomaterials to achieve the repair and regeneration of articular cartilage. Among anti-inflammatory cytokines, TGF-*β* holds promise for future research.

Many members of the TGF-*β* family exist, including TGF-*β*1, 2, and 3. TGF-*β*1 is the most studied, with known functions in cell proliferation, survival, differentiation, and migration. Clinical trials have explored various treatments involving TGF-*β*, such as targeting TGF-*β* in the treatment of osteogenic imperfections [116], utilizing TGF-*β* vaccination in combination with radiation therapy for pancreatic cancer patients [117], observing the impact of TGF-*β*1 on the contraction of esophageal smooth muscle cells in patients with eosinophilic esophagitis [118]. Consequently, researchers began to study whether transporting TGF-*β*1 into the articular cartilage microenvironment through biomaterials can affect the regeneration and repair of damaged cartilage. However, the half-lives of growth factors are very short, and they degenerate quickly, losing their efficacy [119]. Various drugs have been developed to prolong the half-lives of growth factors and control their release. Polymer NPs are the most widely used delivery systems for growth factors, offering varying delivery rates. A novel ginsenoside TGF-*β*1 loaded SF-gelatine porous scaffold (GSTR) has been reported to reduce inflammation, promote cartilage formation, and jointly create a microenvironment conducive to cartilage regeneration [120]. In a study by Yin et al. [121], gelatin microspheres containing TGF-*β*1 were loaded onto a scaffold and injected into the damaged articular cartilage of rabbits, together with adipose stem cells. The scaffold enhanced the differentiation of adipose MSCs and promoted the regeneration of defective cartilage. These results show that combining TGF-*β*1 with biomaterials holds promise as a new and promising treatment for cartilage repair and regeneration. Currently, TGF-*β*1 has entered clinical trials. Genetically engineered chondrocytes expressing TGF-*β*1 were injected into the knees of 54 patients with OA to evaluate their therapeutic effects. It was found that the OA score in the treatment group decreased significantly after the injection [122]. This study provided a sufficient basis for further clinical application of TGF-*β*.

### 4.4. Immune Cells

When cartilage is damaged, the immune microenvironment formed by immune cells plays a critical role in influencing the growth and regeneration of the cartilage. The immune cells involved in cartilage injury and repair include macrophages, osteoclasts, T cells, B cells, natural killer cells (NK cells), and dendritic cells (DCs). In the initial stages of injury, neutrophils are the first immune cells recruited. Neutrophils secrete pro-inflammatory mediators and degradative proteases; these molecules can cause damage to articular cartilage. Neutrophils play a role in activating latent pro-MMP13 by releasing elastase and have been shown to rapidly degrade cartilage collagen *in vitro* [123]. Macrophages, DCs, and NK cells are recruited [124], and they induce chondrocytes apoptosis and ECM degradation. Interferon-gamma (IFN-*γ*) is released after NK cells are activated, and helper T1 (Th1) cells polarize infiltrating macrophages into M1 macrophages. Pro-inflammatory factors secreted by the M1 macrophages interfere with the cartilage differentiation of MSCs. DCs activate Th1 and Th17 cells, leading to cartilage degeneration.

**Figure 6:**
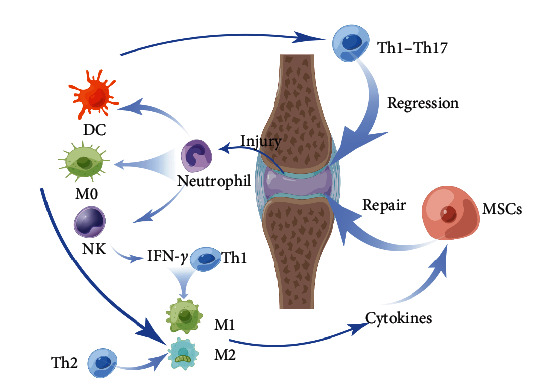
The interaction between immune cells in microenvironment. When cartilage is damaged, neutrophils are first recruited to secrete pro-inflammatory mediators. Macrophages, DCs, and NK cells are subsequently recruited [124]. Th1 cells polarize M0 macrophages into M1 macrophages under the stimulation of IFN-*γ*. DCs induce activation of Th1 and Th17, leading to cartilage degradation. Th2 cells secrete IL-4, which polarizes M0 macrophages into M2 macrophages, inhibits inflammation, and promotes cartilage repair [20].

During the repair process, Th2 cells secrete IL-4, which polarizes macrophages into the M2 phenotype. M2 macrophages secrete anti-inflammatory and chondrogenic factors, inhibit inflammation, and promote cartilage repair. Conversely, M1 macrophages promote tissue fibrosis. Through IL-10 secretion, DCs promote the differentiation of MSCs into chondrocytes and induce regulatory T cells (Tregs) proliferation. Tregs, in turn, promote the expression of IL-10 and TGF-*β*1, thus inhibiting inflammation and promote cartilage formation. Additionally, NK cells stimulate MSCs recruitment and osteoclast differentiation, while neutrophil-derived vesicles induce anti-inflammatory responses (Figure 6).

### 4.5. Cell Senescence and SASP

Aging is a complex process that involves metabolic imbalances and morphological and physiological changes in response to various environmental stimuli. Neighboring cells are affected by different signaling pathways and cytokines that affect normal cell function and lead to multiple diseases, including OA [125], osteoporosis [126], and chronic obstructive pulmonary disease [127].

Senescence-associated secretory phenotype (SASP) is characterized by the secretion of specific bioactive molecules by senescent cells [128]. These molecules can induce physiological responses in the surrounding microenvironment, including inflammation, growth arrest, and tumor formation. Pro-inflammatory cytokines, such as IL-6, IL-17, and TNF-*α* [51, 129], can disrupt the balance of the joint microenvironment, leading to OA and ECM degradation. The SASP is expressed in chondrocytes, osteoblasts, and synovial fibroblasts. These cells show some common characteristics of senescence, such as telomere erosion and increased expression of *p53*, cyclin-dependent kinases, *p21* and *p16*. These changes are attributed to increased levels of ROS caused by mitochondrial dysfunction and the increase in heterochromatin levels associated with aging [42].

Joint tissues are prone to aging and decline over time, with an increased number of aging chondrocytes and synovial fibroblasts [130]. One of the characteristics of OA is an increase in the number of senescent cells. Joint injuries promote chondrocyte senescence and cartilage degeneration [131]. Senescence can induce metabolic remodeling within cells and significantly influence the occurrence and progression of OA. The SASP can secrete bioactive molecules into the surrounding environment to cause inflammation and tumor formation [129]. The expression of *p16*, a biomarker of cell senescence, increases with age. Inhibiting the expression of *p16* in cells has been shown to extend the lifespan of mice. Chondrocytes with high expression of *p16* tend to secrete more ECM catabolic factors, destroying the cartilage [132], an essential factor affecting the microenvironment of articular cartilage.

Senescent cells exhibit the characteristics of SASP and secrete cytokines into the cartilage microenvironment, affecting the repair and regeneration of articular cartilage. Therefore, strategies aimed at inducing apoptosis of senescent cells and inhibiting SASP factors hold promise for the treatment of bone-related diseases in the future, warranting further investigation.

In an animal experiment, the articular cartilage of mice was traumatized, and transgenic technology was used to remove senescent cells from the articular cartilage. The progression of posttraumatic OA was significantly lower than in the group where the senescent cells were removed compared to the control group [131]. There has been growing interest in aging inhibitors in recent years, with different aging inhibitors discovered during this period. In one study, aging upregulates anti-apoptotic genes, such as those in the BCL-2 and PI3-AKT signaling pathways [133]. The mechanism of action of antiaging drugs is by inhibiting these activation pathways and inducing apoptosis in aging cells.

Some known antiaging drugs include Navitoclax, Dasatinib, Quercetin, UBX0101, Fenofibrate, and Fisetin. These drugs are currently undergoing clinical trials for the treatment of various diseases. Navitoclax and Dasatinib are used to treat acute lymphoblastic leukemia and lymphocytic lymphoma [134, 135]; UBX0101 is used to treat idiopathic pulmonary fibrosis [136]; Fenofibrate is used to treat primary cholangitis [137]; and Fisetin is used to treat ischemic stroke [138]. The outcomes of these clinical trials provide a basis for the future application of these drugs to treat OA.

Navitoclax has been reported to be an effective antiaging drug and an inhibitor of anti-apoptotic protein BCL-2. Some studies have shown that intragastrically administered 50 mg/kg Navitoclax can eliminate aging hematopoietic stem cells and muscle stem cells from the bone marrow of mice aged by radiation or normal aging, restoring the vitality of normal aging cells [139]. In one study, 5 mg/kg Dasatinib and 50 mg/kg Quercetin enhanced fracture healing in aged mice by regulating the differentiation of osteoblasts and osteoclasts, significantly reducing the expression of aging markers [140]. Furthermore, injecting 1 mM UBX0101 into the joints of mice with articular cartilage injury every 2 days effectively prevented articular cartilage erosion and relieved pain [131]. Fenofibrate has been proven to be an agonist of peroxisome proliferator-activated receptor *α*. Treatment with 50 *μ*M Fenofibrate can induce apoptosis in aging chondrocytes, reduce the number of aging cells, and protect cartilage from damage [141]. In the study of Zheng et al. [142], chondrocytes were pretreated with 10 *μ*M Fisetin for 2 hr before being treated with IL-1*β*. It was found that it could reverse the decrease of anabolism genes and the increase of catabolism genes induced by IL-1*β* stimulation in chondrocytes [142]. Fisetin is undergoing clinical trials for OA to evaluate its effect on reducing the number of senescent cells in articular cartilage regeneration [125].

Among the SASP factors, IL-1*β*, IL-6, TNF-*α*, and MMP13 affect the microenvironment of articular cartilage. Researchers have identified inhibitors targeting these factors to reduce damage to the articular cartilage. In one trial, individuals injected with anti-IL-1*β* antibody Canakinumab showed a notably lower knee arthroplasty rate than the control group [143]. IL-6 expression is also associated with the occurrence and development of arthritis. The IL-6 receptor inhibitor, Tocilizumab, has shown efficacy in treating rheumatoid arthritis and protects the cartilage matrix [144]. The primary function of MMP13 is to degrade the cartilage matrix and type II collagen in the cartilage. Wang et al. [145] injected CL82198, an MMP13 inhibitor, into mice with a meniscal ligament injury and found that this treatment led to the alleviation of OA symptoms, increased levels of type II collagen, and promoted cartilage regeneration.

## 5. Current Stage of Therapeutic Strategies and Future Prospects

The ultimate goal of OA treatment is to prevent cartilage degeneration and address joint dysfunction. Conventional treatment often involves postoperative exercise and physiotherapy to promote anabolism of the articular cartilage, which may be achieved by activating the TRPV4 channel. However, some patients with OA are unable to exercise due to severe joint injury. Therefore, the use of a TRPV4 activator could offer a solution. Additionally, patients with OA can have hyaluronic acid injections into the joint cavity to enhance lubrication, reduce friction, and decrease local inflammation [146]. However, these injections only provide short-term pain relief and are expensive [147]. Corticosteroid injections are another option for pain management, but like hyaluronic acid, they offer temporary pain relief [146]. These treatments are typically conservative measures employed after disease progression. Research into drugs for the treatment of OA is ongoing; however, their potential application is limited by the presence of toxic side effects [148].

Given the limitations of current OA treatment, we propose a strategy focused on the articular cartilage microenvironment. Piezo1 and Piezo2 are activated during injury. These ion channels experience high mechanical stress, disrupting the balance of Ca^2+^ inside and outside the cell, thus accelerating inflammation. In OA, where articular cartilage is injured, Piezo1 and Piezo2 activation occurs. Hence, inhibiting the opening of their channels is a potential direction of OA treatment in the future. GsMTx4 is an inhibitor of Piezo1 and Piezo2 and has been used to treat Duchenne muscular dystrophy. Intravenous injection of GsMTx4 in cardiac ischemia reduces the infarct area, reduces arrhythmia, and increases cardiac output [149]. GsMTx4 treatment had little effect on the normal group and was only active under pathological conditions. Current research on GsMTx4 highlights its significant potential in treating articular cartilage damage in both *in vitro* and *in vivo* models. Future efforts should focus on *in vivo* animal model experiments on the chondroprotective effects of GsMTx4, including determining appropriate intra-articular injection doses and observing prolonged use of GsMTx4 for potential side effects.

TGF-*β* has shown promise in stimulating cartilage ECM synthesis and promoting articular cartilage regeneration. However, TGF-*β* injection can also cause adverse effects. Chondrocytes in cartilage do not come into direct contact with other cells. The nonvascular characteristics of chondrocytes depend on the signals received by their direct environment. This makes it very difficult to target cartilage without involving other tissues. This means that supplementation of TGF-*β* in joints can also lead to reactions in other tissues that come into contact with synovial fluid [150]. The synovial tissue in articular joints is susceptible to TGF-*β* induced fibroplasias and fibrosis. Therefore, multiple injections of TGF-*β* induced synovial fibrosis in the knee joints of mice [109]. TGF-*β* can also induce osteophytes similar to those found in OA [151]. TGF-*β* expression and active TGF-*β* signal transduction are found to be highly expressed in osteophytes of OA, indicating that TGF-*β* plays a role in OA-induced osteophytes. Additionally, TGF-*β* overexpression has been linked to tumorigenesis and cytotoxicity, limiting its clinical application [152]. Future approaches may involve inhibiting TGF-*β* expression or its downstream signaling pathways at sites with adverse side effects. Moreover, careful dose determination is necessary to prevent the promotion of tumorigenesis and reduce cytotoxicity when using TGF-*β*.

Articular cartilage resides in a hypoxic microenvironment, and avascularity and maintenance of hypoxia are essential for chondrocyte homeostasis. When OA occurs, this hypoxia is disrupted, which leads to an imbalance in the anabolism and catabolism of articular cartilage, thus aggravating OA. HIF-1 is an important gene for maintaining hypoxia and regulating articular cartilage metabolism. In recent years, biomedical applications based on magnesium ions have shown great transformation potential in orthopedics. Magnesium ions can promote the anabolism of articular cartilage through HIF-1*α* [153]. However, regulating the expression of HIF-1 may cause serious side effects because the signal pathway of HIF-1 interferes with other signal pathways, necessitating further exploration.

Aging is the most critical factor in the induction of OA, with a higher incidence in older individuals. As humans age, the population of senescent cells rises, coinciding with a slowdown in cell proliferation and diminished tissue regeneration capacity. Therefore, aging is implicated in the pathogenesis and progression of many diseases. Senescent cells have been shown to promote joint inflammation, causing damage to the articular cartilage [154]. Many antiaging drugs have been developed recently, such as UBX0101, which aims to eliminate aging cells and treat OA. However, no significant difference was observed between the treatment and placebo groups in clinical trials [155]. This raises uncertainty about the efficacy of senescent cell removal in treating OA. Future research should focus on extending treatment duration, as OA management typically requires long-term intervention. While drugs targeting the articular cartilage microenvironment show promise in treating OA, their delivery efficiency to the cartilage tissue after systemic administration is deficient. Direct injection of the drug into the articular cavity is hindered by clearance via the lymphatic system, limiting drug retention in the joint. Therefore, establishing effective drug delivery systems is crucial. Some studies suggest using chondroitin sulfate-derived platinum NPs, which are simple, environmentally friendly, and nontoxic to chondrocytes [156]. These data indicate that this green nanomaterial can be used as a carrier for OA treatment requiring targeted drug delivery.

## 6. Conclusion

The advancements in regeneration methods for the treatment of OA are promising, with ongoing improvements in safety, pain relief, functional enhancement, and cartilage regeneration. Researchers have increasingly focused on the cartilage microenvironment in recent years, recognizing its significance in OA treatment. Altering factors, such as mechanical force, partial pressure of oxygen, cytokines, and senescent cells within the articular cartilage microenvironment, is a promising treatment for OA that promotes the repair and regeneration of damaged cartilage in the future. Despite some treatments having side effects and requiring further clinical trials, ongoing research offers hope for overcoming OA.

## Data Availability

No data were used or generated during this study.
